# Racial/Ethnic Disparities in Financial Hardship During the First Year of the Pandemic

**DOI:** 10.1089/heq.2022.0196

**Published:** 2023-08-30

**Authors:** Alia Alhomsi, Stephanie M. Quintero, Stephanie Ponce, Izabelle Mendez, Anita L. Stewart, Anna Maria Napoles, Paula D. Strassle

**Affiliations:** ^1^ Division of Intramural Research, National Institute on Minority Health and Health Disparities, National Institute of Health, Bethesda, Maryland, USA.; ^2^ Center for Aging in Diverse Communities, Institute for Health & Aging, University of California San Francisco, San Francisco, California, USA.

**Keywords:** financial hardship, lost income, unemployment, housing insecurity, food insecurity, racial–ethnic disparities

## Abstract

**Introduction:**

The economic impact of the COVID-19 pandemic has been substantial, yet little is known about the financial effects resulting from lost employment or financial hardship racial–ethnic disparities.

**Methods:**

We conducted a nationally representative, online survey of 5500 English- and Spanish-speaking American Indian/Alaska Native, Asian, Black/African American, Native Hawaiian/Pacific Islander, Latino, White, and multiracial adults, from December 2020 to February 2021. Six financial hardship domains were measured (lost income, debt, unmet expenses, unmet health care expenses, housing insecurity, and food insecurity). Prevalence of financial hardship among each racial-ethnic group was estimated using multivariable Poisson regression.

**Results:**

Overall, 70.3% reported experiencing financial hardship; debt (57.6%), lost income (44.5%), and unmet expenses (33.7%) were most common. American Indian/Alaska Native (adjusted prevalence ratio [aPR]=1.19, 95% confidence interval [CI]=1.04 to 1.35), Black/African American (aPR=1.18, 95% CI=1.06 to 1.32), Latino (English-speaking: aPR=1.15, 95% CI=1.01 to 1.31; Spanish-speaking: aPR=1.27, 95% CI=1.12 to 1.45), and Native Hawaiian/Pacific Islander (aPR=1.21, 95% CI=1.06 to 1.38) adults were more likely to experience financial hardship, compared with White adults. American Indian/Alaska Native, Black/African American, Spanish-speaking Latino, and Native Hawaiian/Pacific Islander adults were also more likely to report hardship in almost all hardship domains (e.g., housing insecurity: aPRs=1.37–1.91).

**Conclusions:**

Racial/ethnic minorities were more likely to experience financial hardship during the pandemic. The prevalence of lost income was similar across most racial/ethnic groups, suggesting that preexisting wealth disparities led to some groups being less able to handle the economic shocks caused by the COVID-19 pandemic. Financial hardship may be underestimated for communities without English or Spanish fluency. Without intervention, financial hardship will likely exacerbate wealth disparities in the United States.

## Introduction

During the COVID-19 pandemic, racial/ethnic minority households have experienced worse economic fallout and have been disproportionately affected by unemployment and reduced wages compared with White households.^1,2^ The U.S. unemployment rate peaked in April 2020 at 14.8%, having just recently returned to prepandemic levels in July 2022.^3^ At its peak in April 2020, Latino (18.5%), Black (16.7%), and Asian (14.9%) workers experienced higher unemployment rates compared with White workers (14.1%).^4^ This increase in unemployment rates early in the pandemic has likely intensified income and wealth disparities through the loss of health insurance, income, and savings,^1^ pushing lower income racial/ethnic minorities further into poverty.

Even before the COVID-19 pandemic, lower income racial/ethnic minority households and individuals faced higher levels of financial hardship due to racial discrimination and structural barriers in education, housing, employment, and health care.^2,5^ COVID-related discrimination and violence, particularly among Asian communities, could also exacerbate the impacts of financial hardship during the pandemic.^6,7^

With financial hardships exacerbated by the COVID-19 pandemic, racial/ethnic minority families often sacrificed paying for food, medical care, and housing to make ends meet.^2,8^ For instance, one study among adults ages >50 years old found that Black/African American and Latino adults reported more difficulty paying for basic needs and expenses such as food, bills, health care, medical care, and housing during the first year of the pandemic than White adults.^9^ Other studies have found that almost half (49.3%) of Asian adults would be unable to pay for an unexpected expense using cash or savings^10^ and that 60% of Native Hawaiian/Pacific Islander households experienced a loss of income during the first year of the pandemic.^11^ However, additional studies investigating financial hardship during the pandemic among racial/ethnic minority groups, and how financial hardship is experienced (e.g., food insecurity, unmet health care needs) across race–ethnicity is still needed.

To date, no study has simultaneously assessed financial hardship across multiple domains, and few have used nationally representative data or included all major racial/ethnic groups. Thus, the purpose of this study was to (1) describe the burden of financial hardship during the first year of the COVID-19 pandemic across six domains (*lost income*, *unmet expenses*, *debt*, *unmet health care expenses*, *housing insecurity*, and *food insecurity*) and (2) identify racial/ethnic differences in financial hardship during the pandemic, using a diverse, nationally representative sample of English and Spanish speakers living in the United States.

## Methods

### Study population

For this study we used the COVID-19's Unequal Racial Burden (CURB) survey, which was administered online in English and Spanish (Latino participants only) between December 8, 2020, and February 17, 2021, by YouGov, a consumer research firm based in Palo Alto, CA, which uses a proprietary, opt-in survey panel comprising over 1.8 million U.S. residents to conduct nationally representative surveys. Panel members are recruited through a variety of methods to ensure diversity, and then were matched to a theoretical target sample. For this study, the target sample was drawn from the 2018 American Community Survey 1-year sample data, and included 1000 Asian, 1000 Black/African American, 1000 Latino (including 500 Spanish-speaking), 1000 White, 500 American Indian/Alaska Native, 500 Native Hawaiian/Pacific Islander, and 500 multiracial adults ≥18 years of age (*n*=5500 total).

Matched panel members who completed the online survey were then weighted to obtain nationally representative cohorts within each racial/ethnic group (e.g., Asian participants were weighted to represent all Asians living in the United States). The CURB survey development and sampling design details have been previously described.^12^

The National Institutes of Health (NIH) Office of IRB Operations determined that this study does not qualify as human subjects research because data were deidentified (IRB Number: 000166).

The CURB survey included 12 questions relating to financial hardship, the majority of which were adapted from the *All of Us* COVID-19 Participant Experience (COPE) Survey.^13^ Participants were asked if they experienced a time during the pandemic when they did not have enough money to: (1) meet daily needs, (2) pay monthly bills, (3) pay for health care they needed, (4) pay for medications, and (5) pay rent, mortgage, or other housing costs. They were also asked if there was a time when they were hungry but did not eat because there was not enough money for food, and if there was a time when they did not have a regular place to live. The survey included several additional questions about employment changes due to the pandemic (hours reduced, lost job), lost income during the pandemic, lost savings during the pandemic, increased debt during the pandemic, and lost health insurance during the pandemic. Complete data on financial hardship questions were available for >99% of the entire cohort (*n*=5495).

Using these 12 questions, we conceptualized and scored 6 domains of financial hardship: (1) *lost income*, (2) *debt*, (3) *unmet expenses* (general needs), (4) *unmet health care expenses*, (5) *housing insecurity*, and (6) *food insecurity* (Table 1). These domains capture loss of employment and income during the pandemic along with the potential cascade of effects resulting from lost wages and employment. This novel measure allowed us to capture a more comprehensive landscape of financial hardship during the pandemic than that provided by examining lost income alone, as well as assess the differences in the prevalence and relative impact of specific consequences of lost income. A full breakdown of all 12 financial hardship questions and responses are available in Supplementary Table S1.

**Table 1.** Domains and Definitions of Financial Hardship During the Pandemic

Domain	Definition: since the start of the pandemic …	Scoring
*Lost income*	Lost job or business, had hours reduced, or lost work-related income	1=Lost job or business; hours have been reduced; lost work-related income 0=None of these
*Unmet expenses*	Not enough money to meet daily needs, not enough money to pay monthly bills	1=Did not have enough money to meet your daily needs; did not have enough money to pay for your monthly bills 0=None of these
*Debt*	Used all/most of savings or had no savings before pandemic, gone into debt or increased debt	1=Used up all or most of your savings; had no savings before the pandemic; gone into debt or debt has increased 0=None of these
*Unmet health care expenses*	Not enough money to pay for health care, not enough money to pay for medications, lost health insurance	1=Did not have enough money to pay for the health care you needed; did not have enough money to pay for your medications; lost your health insurance 0=None of these
*Housing insecurity*	No regular place to live, not enough money to pay rent, mortgage, or housing costs	1=Did not have a regular place to live; did not have enough money to pay your rent, mortgage, or other housing costs 0=None of these
*Food insecurity*	Hungry but didn't eat because not enough money for food	1=Hungry but didn't eat because there wasn't enough money for food 0=None of these

Financial hardship during the pandemic was operationalized four ways. First, a domain-specific (e.g., *lost income*) dichotomous measure was derived, with 1=reporting that hardship, and 0=not reporting it. Second, an overall financial hardship index was calculated by counting the total number of domains that each participant reported experiencing (range 0–6). Third, based on the index score, we created categories of substantial (4–6 hardship), some (2–3), a little (1), or no (0) hardship. Finally, a financial hardship was dichotomized into any (≥1) hardship versus no (0) hardship.

Participants were also asked “Which one of the following would you say best represents your race/ethnicity?” with response options of Latino or Hispanic, American Indian or Alaska Native, Asian, Black or African American, Native Hawaiian or Pacific Islander, White, or multiracial. Latino participants were further stratified by their survey language preference (English vs. Spanish). Asian, Latino, and Native Hawaiian/Pacific Islander participants were additionally asked about heritage/country of origin to further stratify the racial/ethnic group.

### Statistical analyses

Differences in financial hardship during the pandemic among racial/ethnic groups were assessed using descriptive statistics. Multivariable linear regression, adjusting for age (categorized as 18–29, 30–39, 40–49, 50–59, 60–69, and ≥70 years old), gender (male, female, and transgender/nonbinary), highest education level (less than high school graduate, high school graduate, some college/vocational school, and college graduate or more), and self-reported physical health (poor/fair, good/very, good/excellent), was used to estimate the association between race/ethnicity and the financial hardship index (treated as continuous). Multivariable Poisson regression with robust variance estimators,^14^ adjusting for the same potential confounders listed above, was used to estimate the associations between race/ethnicity and each financial hardship domain, as well as experiencing any financial hardship.

All analyses were performed in SAS version 9.4 (SAS, Inc., Cary, NC). All analyses were weighted to produce nationally representative estimates within each racial/ethnic group and counts were rounded for interpretation.

## Results

There were 5804 online survey respondents (response rate 20.0%) that were matched down to a sample of 5500 to produce the final, weighted dataset. Demographics, stratified by race/ethnicity, are reported in Supplementary Table S2. Overall, 70.3% (*n*=3859) of all participants experienced “any” financial hardship during the first year of the pandemic in at least one domain. Spanish-speaking Latino participants had the highest prevalence of “any” financial hardship during the pandemic (87.3%), followed by Native Hawaiian/Pacific Islander (79.2%), American Indian/Alaska Native (76.8%), and Black/African American (76.4%) adults (Table 2). Over half (57.6%) of participants reported *debt*, and 44.5% reported *lost income* (Table 2). Spanish-speaking Latino participants were more likely than other groups to report *debt* (80.7% vs. 38.5–66.4%) and *lost income* (73.1% vs. 34.9–51.9%).

**Table 2.** Prevalence of Financial Hardship During the Pandemic, Stratified by Race/Ethnicity, Weighted to Be Nationally Representative Within Racial/Ethnic Groups, December 2020 to February 2021

	Overall	American Indian/Alaskan Native	Asian	Black/African American	Latino		Hawaiian/Pacific Islander	White	Multiracial	
					**English**	**Spanish**				
Total, *N*	5492	500	999	998	495	504	496	999	500	
Financial hardship domains, *n* (%)										
Lost income^a^	2448 (44.5)	223 (44.6)	393 (39.3)	428 (42.9)	210 (42.4)	368 (73.1)	259 (51.9)	349 (34.9)	217 (43.4)	
Unmet expenses^b^	1854 (33.7)	189 (37.8)	194 (19.4)	419 (41.9)	180 (36.3)	269 (53.3)	221 (44.7)	226 (22.6)	156 (31.2)	
Debt^c^	3165 (57.6)	328 (65.5)	385 (38.5)	643 (64.3)	322 (64.9)	406 (80.7)	332 (66.4)	464 (46.4)	285 (57.1)	
Unmet health care expenses^d^	1250 (22.7)	143 (28.6)	176 (17.6)	247 (24.7)	115 (23.1)	144 (28.6)	137 (27.6)	162 (16.2)	126 (25.3)	
Housing insecurity^e^	1008 (18.3)	110 (22.0)	103 (10.3)	215 (21.5)	92 (18.5)	155 (30.8)	136 (27.4)	109 (10.9)	89 (17.8)	
Food insecurity^f^	707 (12.9)	102 (20.4)	56 (5.6)	157 (15.7)	62 (12.5)	66 (13.1)	102 (20.5)	90 (9.0)	72 (14.5)	
Financial hardship index,^g^ mean (SD)	1.90 (1.8)	2.19 (1.8)	1.31 (1.5)	2.11 (1.8)	1.98 (1.8)	2.80 (1.7)	2.38 (1.9)	1.40 (1.7)	1.89 (1.8)	
Any financial hardship,^g^ *n* (%)	3859 (70.3)	384 (76.8)	586 (58.6)	763 (76.4)	363 (73.3)	440 (87.3)	393 (79.2)	580 (58.1)	350 (70.1)	
Financial hardship type^g^, n (%)										
Substantial hardship (4–6)	1170 (21.3)	130 (25.9)	121 (12.1)	240 (24.0)	111 (22.4)	182 (36.2)	152 (30.5)	131 (13.1)	104 (20.9)	
Some hardship (2–3)	1503 (27.4)	151 (30.2)	207 (20.7)	294 (29.5)	143 (28.8)	198 (39.4)	141 (28.4)	225 (22.6)	143 (28.7)	
Little hardship (1)	1186 (21.6)	104 (20.7)	258 (25.9)	229 (22.9)	109 (22.1)	59 (11.7)	100 (20.2)	224 (22.4)	102 (20.5)	
No hardships (0)	1633 (29.7)	116 (23.2)	413 (41.4)	235 (23.6)	132 (26.7)	64 (12.7)	103 (20.8)	419 (41.9)	150 (29.9)	

Latino participants were stratified by survey language preference.

^a^ Lost income included loss of job or reduced hours, or loss of work-related income.

^b^ Unmet expenses included not having enough money to meet daily needs or not enough money to pay monthly bills.

^c^ Debt included using up all/most of savings, having no savings before the pandemic, or having gone into debt or increased debt during the pandemic.

^d^ Unmet health care expenses included loss of health insurance, not having enough money to pay for health care, and not having enough money to pay for medications.

^e^ Housing insecurity included not having a regular place to live and not having enough money to pay rent, mortgage, or housing costs.

^f^ Food insecurity included being hungry but did not eat because not enough money for food.

^g^ Financial hardship index was calculated by counting the number of domains each participant reported experiencing (range 0–6); financial hardship was categorized as dichotomous (≥1 [any] vs. no hardships) and into categories (substantial [4–6], some [2–3], little [1], and no hardships).

SD, standard deviation.

When stratified further by heritage/country of origin, substantial variation was observed among subgroups of Asian, Latino, and Native Hawaiian/Pacific Islander adults (Supplementary Tables S3–S5). Among Asian adults, Chinese adults (41.7%) were much less likely to experience “any” financial hardship during the pandemic compared with other Asian groups (59.9–72.8%). Among Latino adults, Central American (85.6%), Mexican/Mexican American/Chicano (83.4%), and other Hispanic/Latino/Spanish (79.3%) adults were more likely to experience “any” financial hardship during the pandemic, compared with other groups (69.3–77.9%); Puerto Rican adults had the lowest prevalence (69.3%). Pacific Islander (84.7%) adults reported a higher prevalence of “any” hardship than Native Hawaiian adults (74.7%).

The overall mean number of financial hardship domains experienced during the pandemic was 1.90 (standard deviation [SD] 1.8). Similar to the prevalence of “any” financial hardship during the pandemic, substantial variation in the number of hardship domains across race/ethnicity and heritage/country of origin were observed (Table 2 and Supplementary Tables S3–S5).

After adjustment, American Indian/Alaska Native (adjusted prevalence ratio [aPR]=1.19, 95% confidence interval [CI]=1.04 to 1.35), Black/African American (aPR=1.18, 95% CI=1.06 to 1.32), Latino (English-speaking: aPR=1.15, 95% CI=1.01 to 1.31; Spanish-speaking: aPR=1.27, 95% CI=1.12 to 1.45), and Native Hawaiian/Pacific Islander (aPR=1.21, 95% CI=1.06 to 1.38) adults were more likely to experience “any” financial hardship, compared with White adults (Table 3). No statistically significant differences in “any” hardship were seen between Asian or multiracial adults compared with White adults. Similar results were seen when comparing the number of financial hardship domains (Table 3). All groups (means=1.89–2.80), except for Asian adults (mean=1.31, SD=1.5) reported a higher mean number of financial hardship domains than White adults (mean=1.40, SD=1.7).

**Table 3.** Adjusted Associations Between Race/Ethnicity and Financial Hardship Domains, Weighted to Be Nationally Representative Within Racial/Ethnic Groups, December 2020 to February 2021

	Any hardship	No. of domains
	aPR (95% CI)^a^	Mean difference (95% CI)^a^
American Indian/Alaska Native	1.19 (1.04 to 1.35)	0.44 (0.26 to 0.62)
Asian	0.98 (0.88 to 1.11)	−0.19 (−0.34 to −0.05)
Black/African American	1.18 (1.06 to 1.32)	0.40 (0.26 to 0.55)
Latino		
English-language preference^b^	1.15 (1.01 to 1.31)	0.29 (0.12 to 0.47)
Spanish-language preference^b^	1.27 (1.12 to 1.45)	0.87 (0.69 to 1.06)
Hawaiian/Pacific Islander	1.21 (1.06 to 1.38)	0.59 (0.41 to 0.77)
White	1.0 (Ref.)	1.0 (Ref.)
Multiracial	1.09 (0.95 to 1.24)	0.14 (−0.04 to 0.32)

^a^ Adjusted for age, gender, highest education level, and self-reported physical health.

^b^ Latino participants were stratified based on survey language preference.

aPR, adjusted prevalence ratio; CI, confidence interval.

Racial/ethnic differences were observed also across the specific financial hardship domains (Table 4). Native Hawaiian/Pacific Islander adults were the only group that experienced greater hardship than White adults in all six domains (*lost income* aPR=1.27, 95% CI=1.08 to 1.50; *debt* aPR=1.22, 95% CI=1.06 to 1.41; *unmet expenses* aPR=1.51, 95% CI=1.25 to 1.82; *unmet health care expenses* aPR=1.34, 95% CI=1.06 to 1.68; *housing insecurity* aPR=1.84, 95% CI=1.43 to 2.37; and *food insecurity* aPR=1.54, 95% CI=1.15 to 2.05).

**Table 4.** Adjusted Associations Between Race/Ethnicity and Specific Financial Hardship Domains, Weighted to Be Nationally Representative Within Racial/Ethnic Groups, December 2020 to February 2021

	Lost income	Debt	Unmet needs	Unmet health care needs	Housing insecurity	Food insecurity
	aPR (95% CI)^a^	aPR (95% CI)^a^	aPR (95% CI)^a^	aPR (95% CI)^a^	aPR (95% CI)^a^	aPR (95% CI)^a^
American Indian/Alaska Native	1.15 (0.97 to1.36)	1.21 (1.05 to 1.40)	1.30 (1.07 to 1.58)	1.40 (1.12 to 1.76)	1.56 (1.19 to 2.04)	1.59 (1.19 to 2.12)
Asian	1.03 (0.89 to 1.19)	0.83 (0.72 to 0.95)	0.85 (0.70 to 1.03)	1.03 (0.83 to 1.28)	0.89 (0.67 to 1.16)	0.62 (0.44 to 0.87)
Black/African American	1.07 (0.93 to 1.24)	1.22 (1.08 to 1.37)	1.48 (1.26 to 1.75)	1.26 (1.03 to 1.54)	1.54 (1.22 to 1.94)	1.29 (1.00 to 1.68)
Latino						
English-language preference^b^	1.08 (0.91 to 1.29)	1.24 (1.08 to 1.43)	1.32 (1.08 to 1.60)	1.20 (0.94 to 1.53)	1.37 (1.04 to 1.81)	1.06 (0.76 to 1.46)
Spanish-language preference^b^	1.69 (1.45 to 1.97)	1.42 (1.23 to 1.63)	1.69 (1.41 to 2.04)	1.31 (1.04 to 1.66)	1.91 (1.48 to 2.46)	0.87 (0.63 to 1.21)
Hawaiian/Pacific Islander	1.27 (1.08 to 1.50)	1.22 (1.06 to 1.41)	1.51 (1.25 to 1.82)	1.34 (1.06 to 1.68)	1.84 (1.43 to 2.37)	1.54 (1.15 to 2.05)
White	1.0 (Ref.)	1.0 (Ref.)	1.0 (Ref.)	1.0 (Ref.)	1.0 (Ref.)	1.0 (Ref.)
Multiracial	1.06 (0.89 to 1.26)	1.08 (0.93 to 1.26)	1.11 (0.90 to 1.36)	1.22 (0.97 to 1.55)	1.25 (0.82 to 1.53)	1.12 (0.82 to 1.53)

^a^ Adjusted for age, gender, highest education level, and self-reported physical health.

^b^ Latino participants were stratified based on survey language preference.

American Indian/Alaska Native (*debt* aPR=1.21, 95% CI=1.05 to 1.40; *unmet expenses* aPR=1.30, 95% CI=1.07 to 1.58; *unmet health care expenses* aPR=1.40, 95% CI=1.12 to 1.76; *housing insecurity* aPR=1.56, 95% CI=1.19 to 2.04; and *food insecurity* aPR=1.59, 95% CI=1.19 to 2.12) and Black/African American (*debt* aPR=1.22, 95% CI=1.08 to 1.37; *unmet expenses* aPR=1.48, 95% CI=1.26 to 1.75; *unmet health care expenses* aPR=1.26, 95% CI=1.03 to 1.54; *housing insecurity* aPR=1.54, 95% CI=1.22 to 1.94; and *food insecurity* aPR=1.29, 95% CI=1.00 to 1.68) adults both reported greater hardship than White adults on the same five of six domains.

Spanish-speaking Latino adults also reported greater hardship than White adults on five of six domains, the same domains as American Indian/Alaska Native and Black/African American adults except for one (*lost income* aPR=1.69, 95% CI=1.45 to 1.97; *debt* aPR=1.42, 95% CI=1.23 to 1.63; *unmet expenses* aPR=1.69, 95% CI=1.41 to 2.04; *unmet health care expenses* aPR=1.31, 95% CI=1.04 to 1.66; and *housing insecurity* aPR=1.91, 95% CI=1.48 to 2.46). English-speaking Latino adults reported greater hardship than White adults on three of six domains (*debt* aPR=1.24, 95% CI=1.08 to 1.43; *unmet expenses* aPR=1.32, 95% CI=1.08 to 1.60; and *housing insecurity* aPR=1.37, 95% CI=1.04 to 1.81). Finally, Asian and multiracial adults did not differ from White adults on any financial hardship domain.

## Discussion

Using data from a nationally representative, diverse cohort of English- and Spanish-speaking adults living in the U.S., we found that 58–87% of adults within each racial/ethnic/language group experienced at least one type (any) of financial hardship during the first year of the pandemic. By far the most common hardship across all groups was debt, reported by 58% of participants. The second most common hardship was lost income reported by 45%. Overall, one-third to one-half of respondents reported in descending order debt, lost income, and unmet expenses, pointing to widespread experiences of economic downturn in American households. American Indian/Alaska Native, Black/African American, Spanish-speaking Latino, and Native Hawaiian/Pacific Islander adults were more likely than White adults to report hardship in at least five of six financial hardship domains. Food insecurity and lost income were also notably higher in specific non-White groups.

Our findings revealed striking disparities among Native Hawaiian/Pacific Islander, Spanish-speaking Latino, Black/African American adults, and American Indian/Alaska Native adults, with notable, but fewer disparities among English-speaking Latinos, signaling the potential continuation of long-standing disparities associated with structural and institutional racism.^15–17^ Such structural factors have caused widening racial/ethnic gaps in wealth, unemployment rates, and declines in homeownership, and an inability to build generational wealth, which still have substantial effects on racial/ethnic minority families today^17,18^ and could potentially worsen.

In 2019, White households had eight times the wealth of Black households and five times the wealth of Latino households.^19^ In, 2000 it was estimated that American Indian/Alaska Native households had 8 cents for every dollar held by a White household.^20^ Native Hawaiian families have the lowest mean family income of all major ethnic groups in Hawaii and are presented with unique economic challenges with the higher cost of living in the state.^21^

Consistent with our findings, previous research has also shown that Spanish-speaking Latino individuals and others with limited English proficiency are more vulnerable to economic crises than their English-speaking counterparts.^22,23^ Asian adults with limited English proficiency have also been found to have higher poverty rates and be on Medicaid or uninsured compared with their English-proficient counterparts.^24^ Racial/ethnic minorities are more likely to hold public facing job positions in essential businesses, especially Latinos who are more likely than other groups to work in service or production occupations that often do not offer health insurance coverage.^25^ Compared with White adults, we found that American Indian/Alaska Native, Black/African American, Spanish-speaking Latino, and Native Hawaiian/Pacific Islander adults were all more likely to report having lost their health insurance and having difficulty paying for health care and medications.

Even before the pandemic, American Indian/Alaska Native (22%), Black/African American (11%), Latino (20%), and Native Hawaiian/Pacific Islander (13%) adults were more likely to be uninsured, compared with White adults (8%).^26^ Job losses may have exacerbated insurance disparities among Spanish-speaking Latino and Native Hawaiian/Pacific Islander adults in particular; employer-sponsored insurance is the largest provider of insurance among working-age adults in the United States, and estimates suggest that millions of adults are newly uninsured due to job loss during the pandemic.^27,28^ Studies have also found that racial/ethnic minority adults are more likely to be underinsured, and therefore less likely to be able to afford health care or go into debt/use savings due to high deductibles and poor coverage.^29^

American Indian/Alaska Native, Black/African American, Latino (English- and Spanish-speaking), and Native Hawaiian/Pacific Islander adults were all more likely to report housing insecurity, compared with White adults, especially Spanish-speaking Latino and Native Hawaiian/Pacific Islander. This greater burden may be partially due to Spanish-speaking families having the highest rate of housing-cost burden^23^ and Hawaii having the highest cost of living in the United States.^30^ The housing and eviction crisis during the COVID-19 pandemic has been well documented.^31,32^ However, the pandemic did not create the problem, rather the cost burden of housing—particularly among renters—has been increasing for decades; since 1960, renter incomes have increased by 5%, whereas rent has increased by over 60%.^33^ More than half of Black/African American and Latino renters spend more than 30% of their income on housing.^33^

Food insecurity has also risen dramatically between 2020 and 2021 due to the COVID-19 pandemic, impacting an estimated 42 million people in the United States.^34^ In our analysis, American Indian/Alaska Native, Black/African American, and Native Hawaiian/Pacific Islander adults had higher rates of experiencing food insecurity relative to White adults. Before the pandemic, American Indian and Alaska Native communities already experienced widespread food insecurity along with a disproportionate burden of chronic diet-related diseases.^35^ Drastic changes in dietary patterns through the forced removal from traditional lands and increased reliance on government food programs are major causes of food insecurity among American Indian/Alaska Native individuals,^35^ which may have been exacerbated due to limited resources during the pandemic. Additionally, during the pandemic, among food insecure households, Black/African American households were more likely to report being unable to afford food.^36^

Although not designed to be representative, our estimates of financial hardship across Asian subgroups indicate some differences that require further study and may be masked when looking at these group in aggregate. For example, Filipino and other Asian adults had higher unadjusted prevalence of any financial hardship compared with estimates among Asian (aggregated) and White adults. Other studies have also found that the COVID-19 pandemic has had differential economic impacts among Asian subgroups.^37^

Only conducting the survey in English among Asian participants may also explain why we did not find significant differences in financial hardship between Asian and White adults. For example, while one study of Asian adults in New York City similarly found that 39% of participants reported lost income, they also found that 51% participants reported food insecurity.^38^ This survey was offered in 11 Asian languages in addition to English, and the inclusion of Asian adults with limited English proficiency (in addition to only including Asian adults living in NYC) likely explain why our results differ so drastically.

Taken together, our findings and others highlight the need to collect disaggregated data among Asian adults as well as conducting surveys in Asian languages to get a more complete picture of how this community has been impacted.

We also observed differences across our Latino participants. In addition to differences by language preference (a surrogate for acculturation), Mexican, Central American, and other Latino origin groups tended to report higher rates of financial hardship compared with Puerto Rican, Cuban, and South American adults, which is consistent with previously reported differences in socioeconomic status.^39^

This study has some limitations. First, the survey was administered online, and individuals with limited internet access or familiarity with technology and limited literacy may be less likely to enroll in the YouGov panel or participate in our study. While we did match and weight participants to obtain a nationally representative sample using English- and Spanish-speaking adults, it is possible that some selection bias exists. Given that socioeconomic status is associated with both financial hardship and ability to access and use technology,^40^ it is possible we underestimated the burden of financial hardship during the pandemic.

Second, our CURB survey was only administered in English and Spanish (Latino participants only), which means non-Spanish-speaking individuals with limited English proficiency were less likely to participate. In our study, 12.3% of Asian reported limited English proficiency, which is lower than 2020 national estimates (31.9%).^41^ Prior research has found that the economic impact of the pandemic has been substantial among Asian adults with limited English proficiency,^38^ and our results likely underestimate the burden of financial hardship among the Asian community due to only administering the survey in English.

Our survey also had a relatively low response rate among YouGov panel members (20%). Finally, while our survey was designed to be representative of the major U.S. racial/ethnic groups, stratified results for Asian, Latino, and Native Hawaiian/Pacific Islander adults by heritage/country of origin may not be representative and sample sizes were small in some groups.

## Health Equity Implications

This is one of the first, and most nuanced, assessments of racial/ethnic disparities in financial hardship during the pandemic in a nationally representative, diverse population of English- and Spanish-speaking U.S. adults. Overall, we found that experiencing financial hardship was common (58–87% across racial/ethnic groups), and that Native Hawaiian/Pacific Islander and Spanish-speaking Latino adults were the hardest hit, followed closely by American Indian/Alaska Native, and Black/African American adults. Differences across Asian, Latino, and Native Hawaiian/Pacific Islander subgroups were also seen. The prevalence of lost income was similar across most racial/ethnic groups, suggesting that preexisting wealth disparities led to some groups being less able to handle the economic shocks caused by the COVID-19 pandemic. Without intervention, financial hardship during the pandemic will likely exacerbate economic inequalities and wealth disparities in the United States.

To ensure economic recovery from the pandemic for the most vulnerable, we will need to enact policies that provide economic assistance for the most socioeconomically disadvantaged that includes support for basic necessities, such as employment, housing, food, and health care.

## Supplementary Material

Supplemental data

Supplemental data

Supplemental data

## Abbreviations Used

aPR: adjusted prevalence ratio
CI: confidence interval
CURB: COVID-19's Unequal Racial Burden
SD: standard deviation
