# Supplementary material for: Racial/Ethnic Disparities in Financial Hardship During the First Year of the Pandemic
Source: Health Equity. 2023 Aug 30;7(1):453–61. doi: 10.1089/heq.2022.0196 (PMC10523407; doi:10.1089/heq.2022.0196)
Supplement: Supplemental data [file Suppl_TableS1.docx]

**Supplemental Table 1.** Breakdown of survey questions on financial hardship during the pandemic in the COVID-19’s Unequal Racial Burden (CURB) survey, weighted to be nationally representative within racial/ethnic groups.

|  | **Yes**  **N (%)** | **No**  **N (%)** |
| --- | --- | --- |
| **Lost income** |  |  |
| Since the start of the pandemic, have you lost your job or business? | 2,025 (36.8)^a^ | 3,473 (63.1) |
| Since the start of the pandemic, have you lost any work-related income? | 2,162 (39.3) | 3,336 (60.6) |
| **Unmet expenses** |  |  |
| Since the start of the pandemic, was there ever a time when you did not have enough money to meet your daily needs? | 1,363 (24.7) | 4,133 (75.2) |
| Since the start of the pandemic, was there ever a time when you did not have enough money to pay your monthly bills? | 1,473 (26.8) | 4,022 (73.1) |
| **Debt** |  |  |
| Since the start of the pandemic, have you had to use up all or most of your savings? | 2,899 (52.7)^b^ | 2,601 (47.2) |
| Since the start of the pandemic, have you gone into debt or has your debt increased? | 1,861 (33.8) | 3,639 (66.1) |
| **Unmet healthcare expenses** |  |  |
| Since the start of the pandemic, was there ever a time when you lost your health insurance? | 584 (10.6) | 4,915 (89.3) |
| Since the start of the pandemic, was there ever a time when you did not have enough money to pay for the health care that you needed? | 682 (12.4) | 4,813 (87.5) |
| Since the start of the pandemic, was there ever a time when you did not have enough money to pay for your medications? | 600 (10.9) | 4,895 (89.0) |
| **Housing insecurity** |  |  |
| Since the start of the pandemic, was there ever a time when you did not have enough money to pay your rent, mortgage, or other housing costs? | 933 (16.9) | 4,562 (83.0) |
| Since the start of the pandemic, was there ever a time when you did not have a regular place to live? | 227 (4.1) | 5,268 (95.8) |
| **Food insecurity** |  |  |
| Since the start of the pandemic, was there ever a time when you were hungry but didn’t eat because there wasn’t enough money for food? | 707 (12.8) | 4,788 (87.1) |
| ^a^ 20.2% (n=1,113) reported losing their job and 16.6% (n=912) reported having their hours reduced  ^b^ 33.8% (n=1,864) reported using up all or most of their savings during the pandemic and 18.8% of participants (n=1,035) reported having no savings before the pandemic | | |
