# Supplementary material for: Racial/Ethnic Disparities in Financial Hardship During the First Year of the Pandemic
Source: Health Equity. 2023 Aug 30;7(1):453–61. doi: 10.1089/heq.2022.0196 (PMC10523407; doi:10.1089/heq.2022.0196)
Supplement: Supplemental data [file Suppl_TableS2.docx]

**Supplemental Table 2.** Demographics and other participant characteristics, weighted to be nationally representative within each racial/ethnic group for English and Spanish speakers, among participants of the COVID-19’s Unequal Racial Burden (CURB) survey, December 2020 – February 2021, n=5,550.

|  | **Overall** | **American Indian/ Alaska Native** | **Asian** | **Black/African American** | **Latino** | **Hawaiian/ Pacific Islander** | **White** | **Multiracial** |
| --- | --- | --- | --- | --- | --- | --- | --- | --- |
| **Total^a^, N** | 5,500 | 500 | 1000 | 1000 | 1000 | 500 | 1000 | 500 |
| **Age, years, median (IQR)** | 42 (29, 58) | 43 (29, 60) | 42 (30, 56) | 41 (29, 57) | 39 (28, 54) | 40 (29, 54) | 52 (34, 64) | 35 (25, 52) |
| **Gender, n (%)** |  |  |  |  |  |  |  |  |
| Male | 2,588 (47.1) | 231 (46.2) | 456 (45.9) | 472 (47.2) | 490 (49.0) | 236 (47.2) | 475 (47.6) | 228 (45.6) |
| Female | 2,771 (50.5) | 245 (49.1) | 525 (52.8) | 513 (51.3) | 492 (49.2) | 250 (50.0) | 509 (50.9) | 238 (47.5) |
| Non-binary^b^ or transgender | 133 (2.4) | 23 (4.6) | 13 (1.3) | 15 (1.5) | 18 (1.8) | 13 (2.6) | 15 (1.5) | 34 (6.8) |
| **Health insurance, n (%)** |  |  |  |  |  |  |  |  |
| Any private | 2,384 (43.6) | 151 (31.3) | 574 (58.8) | 323 (33.5) | 294 (31.1) | 189 (39.4) | 506 (53.0) | 244 (50.3) |
| Public insurance only | 1,953 (35.7) | 227 (47.2) | 253 (25.9) | 422 (43.6) | 282 (29.8) | 204 (42.5) | 327 (34.2) | 167 (34.5) |
| Uninsured | 1,137 (20.8) | 103 (21.5) | 148 (15.2) | 221 (22.9) | 371 (39.2) | 88 (18.2) | 122 (12.8) | 73 (15.1) |
| **Immigration status, n (%)** |  |  |  |  |  |  |  |  |
| US-born citizen | 4,276 (77.8) | 485 (97.2) | 515 (51.5) | 907 (90.7) | 494 (49.4) | 443 (88.7) | 977 (97.8) | 456 (91.1) |
| Foreign-born citizen\legal resident | 946 (17.2) | 14 (2.8) | 433 (43.3) | 83 (8.3) | 301 (30.1) | 50 (10.1) | 21 (2.1) | 43 (8.6) |
| Undocumented | 275 (5.0) | 0 (0.0) | 52 (5.2) | 10 (1.0) | 204 (20.4) | 6 (1.2) | 1 (0.1) | 1 (0.3) |
| **Limited English proficiency^c^, n (%)** | 618 (11.2) | 29 (5.9) | 123 (12.3) | 48 (4.8) | 357 (35.7) | 33 (6.6) | 22 (2.2) | 6 (1.1) |
| **Education, n (%)** |  |  |  |  |  |  |  |  |
| Less than high school | 498 (9.1) | 57 (11.4) | 40 (4.0) | 68 (6.8) | 188 (18.8) | 41 (8.3) | 55 (5.5) | 49 (9.8) |
| High school/GED | 1,791 (32.6) | 192 (38.5) | 229 (22.9) | 375 (37.5) | 387 (38.7) | 196 (39.2) | 291 (29.1) | 120 (24.0) |
| Some college/vocational | 1,690 (30.7) | 181 (36.2) | 215 (21.5) | 351 (35.1) | 274 (27.4) | 178 (35.7) | 312 (31.2) | 180 (36.0) |
| College graduate or more | 1,520 (27.6) | 70 (13.9) | 516 (51.6) | 207 (20.7) | 151 (15.1) | 84 (16.8) | 342 (34.2) | 151 (30.1) |
| **Family annual income^d^, n (%)** |  |  |  |  |  |  |  |  |
| <$20,000 | 1,095 (22.8) | 123 (26.7) | 106 (12.4) | 292 (33.3) | 247 (28.1) | 110 (24.8) | 130 (15.2) | 86 (19.8) |
| $20,000-$59,999 | 1,921 (40.0) | 193 (41.9) | 281 (32.8) | 346 (39.4) | 423 (48.2) | 165 (37.1) | 336 (39.2) | 177 (41.0) |
| $60,000-$99,999 | 974 (20.3) | 81 (17.5) | 229 (26.7) | 137 (15.6) | 139 (15.9) | 108 (24.3) | 181 (21.1) | 100 (23.1) |
| ≥$100,000 | 818 (17.0) | 64 (13.9) | 241 (28.1) | 103 (11.8) | 69 (7.9) | 61 (13.7) | 211 (24.6) | 69 (16.0) |
| Prefer not to say | 692 | 39 | 144 | 122 | 122 | 56 | 142 | 68 |
| **Married^e^, n (%)** | 2,551 (46.4) | 238 (47.6) | 507 (50.7) | 318 (31.8) | 533 (53.3) | 248 (49.6) | 522 (52.2) | 186 (37.2) |
| **Census division, n (%)** |  |  |  |  |  |  |  |  |
| New England | 160 (2.9) | 7 (1.5) | 53 (5.3) | 17 (1.7) | 28 (2.8) | 6 (1.2) | 32 (3.2) | 17 (3.4) |
| Middle Atlantic | 622 (11.3) | 14 (2.7) | 163 (16.3) | 131 (13.1) | 100 (10.0) | 8 (1.7) | 152 (15.2) | 54 (10.7) |
| East North Central | 554 (10.1) | 41 (8.1) | 83 (8.3) | 134 (13.4) | 58 (5.8) | 18 (3.6) | 159 (15.9) | 61 (12.2) |
| West North Central | 224 (4.1) | 35 (7.1) | 23 (2.3) | 31 (3.1) | 18 (1.8) | 8 (1.5) | 94 (9.4) | 16 (3.2) |
| South Atlantic | 1,070 (19.5) | 46 (9.2) | 142 (14.2) | 348 (34.8) | 183 (18.3) | 43 (8.6) | 210 (21.0) | 99 (19.8) |
| East South Central | 252 (4.6) | 19 (3.8) | 10 (1.0) | 117 (11.7) | 14 (1.4) | 7 (1.4) | 63 (6.3) | 22 (4.4) |
| West South Central | 668 (12.2) | 76 (15.3) | 79 (7.9) | 139 (13.9) | 204 (20.4) | 20 (4.1) | 99 (9.9) | 51 (10.2) |
| Mountain | 555 (10.1) | 113 (22.6) | 71 (7.1) | 29 (2.9) | 97 (9.7) | 91 (18.2) | 93 (9.3) | 60 (12.0) |
| Pacific | 1,394 (25.4) | 149 (29.8) | 377 (37.7) | 54 (5.4) | 297 (29.7) | 298 (59.7) | 99 (9.9) | 120 (24.0) |
| **Residence urbanicity, n (%)** |  |  |  |  |  |  |  |  |
| Big city | 1,433 (26.8) | 99 (19.9) | 265 (26.5) | 344 (34.9) | 376 (37.8) | 98 (19.5) | 130 (15.1) | 122 (24.3) |
| Smaller city | 1,022 (19.1) | 92 (18.4) | 161 (16.1) | 193 (19.6) | 266 (26.8) | 89 (17.7) | 134 (15.5) | 87 (17.4) |
| Suburban area | 1,664 (31.1) | 101 (20.2) | 415 (41.6) | 300 (30.4) | 228 (23.0) | 153 (30.7) | 285 (32.9) | 182 (36.3) |
| Small town | 659 (12.3) | 94 (18.8) | 106 (10.6) | 83 (8.4) | 67 (6.7) | 98 (19.6) | 150 (17.4) | 62 (12.3) |
| Rural | 566 (10.6) | 114 (22.8) | 53 (5.3) | 67 (6.8) | 56 (5.7) | 62 (12.5) | 166 (19.2) | 48 (9.5) |
| Abbreviations: IQR, interquartile range  ^a^ Online survey of US adults, weighted to be nationally representative within each racial/ethnic group; due to rounding percentages may not sum to 100%  ^b^ Includes individuals who identified as non-binary, gender queer, gender fluid, other, and none  ^c^ Limited English proficiency was defined as speaking English "not at all", "poorly", and "fairly well"  ^d^ Collected by YouGov at enrollment into panel and updated every 6 months  ^e^ Collected by YouGov at enrollment into panel and updated every 12 months | | | | | | | | |
