# Supplementary material for: Racial/Ethnic Disparities in Financial Hardship During the First Year of the Pandemic
Source: Health Equity. 2023 Aug 30;7(1):453–61. doi: 10.1089/heq.2022.0196 (PMC10523407; doi:10.1089/heq.2022.0196)
Supplement: Supplemental data [file Suppl_TableS3-S5.docx]

**Supplemental Table 3.** Prevalence of financial hardship during the pandemic among Asian adults, stratified by heritage, weighted to be nationally representative within racial/ethnic groups, December 2020-Februrary 2021.

|  | **Asian Indian** | | **Chinese** | | **Filipino** | | **Japanese** | | **Other Asian** | |
| --- | --- | --- | --- | --- | --- | --- | --- | --- | --- | --- |
| **Total, N** | 175 |  | 274 |  | 161 |  | 131 |  | 258 |  |
| **Financial hardship domains, n (%)** |  |  |  |  |  |  |  |  |  |  |
| Lost income^a^ | 81 | (46.6) | 89 | (32.3) | 79 | (49.1) | 29 | (22.1) | 115 | (44.6) |
| Unmet expenses^b^ | 28 | (16.2) | 24 | (8.6) | 55 | (34.4) | 31 | (23.7) | 55 | (21.4) |
| Debt^c^ | 70 | (40.1) | 54 | (19.9) | 85 | (52.8) | 60 | (45.4) | 116 | (44.9) |
| Unmet healthcare expenses^d^ | 29 | (16.7) | 32 | (11.6) | 39 | (24.5) | 25 | (19.1) | 50 | (19.3) |
| Housing insecurity^e^ | 18 | (10.4) | 15 | (5.5) | 22 | (13.6) | 10 | (7.5) | 38 | (14.6) |
| Food insecurity^f^ | 12 | (6.7) | 4 | (1.5) | 11 | (6.7) | 7 | (5.6) | 22 | (8.6) |
| **Financial hardship index^g^, mean (SD)** | 1.37 | (1.5) | 0.79 | (1.2) | 1.81 | (1.7) | 1.24 | (1.5) | 1.53 | (1.6) |
| **Any financial hardship^g^, n (%)** | 105 | (59.9) | 114 | (41.7) | 117 | (72.8) | 83 | (63.5) | 167 | (64.6) |
| **Financial hardship type^g^, n (%)** |  |  |  |  |  |  |  |  |  |  |
| Substantial hardship (4-6) | 23 | (12.9) | 14 | (5.3) | 33 | (20.7) | 10 | (7.4) | 41 | (15.7) |
| Some hardship (2-3) | 41 | (23.3) | 39 | (14.1) | 41 | (25.3) | 30 | (22.7) | 57 | (22.1) |
| Little hardship (1) | 41 | (23.7) | 61 | (22.3) | 43 | (26.7) | 44 | (33.3) | 69 | (26.8) |
| No hardships (0) | 70 | (40.1) | 160 | (58.3) | 44 | (27.2) | 48 | (36.5) | 92 | (35.4) |
| ^a^ Lost income included loss of job or reduced hours, or loss of work-related income  ^b^ Unmet expenses included not having enough money to meet daily needs or not enough money to pay monthly bills  ^c^ Debt included using up all/most of savings, having no savings before the pandemic, or having gone into debt or increased debt during the pandemic  ^d^ Unmet healthcare expenses included loss of health insurance, not having enough money to pay for healthcare, and not having enough money to pay for medications  ^e^ Housing insecurity included not having a regular place to live and not having enough money to pay rent, mortgage, or housing costs  ^f^ Food insecurity included being hungry but didn’t eat because not enough money for food  ^g^ Financial hardship index was calculated by counting the number of domains each participant reported experiencing (range 0-6); financial hardship was categorized as dichotomous (≥1 [any] vs. no hardships) and into categories (substantial [4-6], some [2-3], little [1], and no hardships) | | | | | | | | | | |

**Supplemental Table 4.** Prevalence of financial hardship during the pandemic among Latino adults, stratified by heritage, weighted to be nationally representative within racial/ethnic groups, December 2020-Februrary 2021.

|  | **Mexican/**  **Mexican American/**  **Chicano** | | **Puerto**  **Rican** | | **Cuban/ Dominican** | | | **Central American** | | **South**  **American** | | **Other Latino** | |
| --- | --- | --- | --- | --- | --- | --- | --- | --- | --- | --- | --- | --- | --- |
| **Total, N** | 528 |  | 114 |  | 85 |  | | 80 |  | 99 |  | 94 |  |
| **Financial hardship domains, n (%)** |  |  |  |  |  |  | |  |  |  |  |  |  |
| Lost income^a^ | 334 | (63.3) | 31 | (27.4) | 45 | (53.0) | | 54 | (67.3) | 52 | (52.8) | 62 | (66.3) |
| Unmet expenses^b^ | 238 | (45.2) | 50 | (43.7) | 33 | (38.7) | | 40 | (50.8) | 37 | (37.2) | 50 | (53.4) |
| Debt^c^ | 398 | (75.4) | 73 | (63.6) | 59 | (70.0) | | 63 | (78.6) | 67 | (67.5) | 68 | (72.7) |
| Unmet healthcare expenses^d^ | 142 | (27.0) | 34 | (29.6) | 15 | (17.3) | | 16 | (19.9) | 22 | (22.6) | 30 | (31.7) |
| Housing insecurity^e^ | 138 | (26.1) | 27 | (23.2) | 17 | (20.5) | | 22 | (27.9) | 21 | (21.6) | 22 | (23.0) |
| Food insecurity^f^ | 70 | (13.2) | 19 | (16.7) | 12 | (13.6) | | 7 | (9.3) | 8 | (8.0) | 13 | (13.3) |
| **Financial hardship index^g^, mean (SD)** | 2.50 | (1.8) | 2.04 | (1.9) | 2.13 | (1.8) | | 2.54 | (1.6) | 2.10 | (1.6) | 2.61 | (1.9) |
| **Any financial hardship^g^, n (%)** | 440 | (83.4) | 80 | (69.3) | 66 | (77.9) | | 68 | (85.6) | 75 | (75.8) | 75 | (79.3) |
| **Financial hardship type^g^, n (%)** |  |  |  |  |  |  |  | |  |  | |  |  |
| Substantial hardship (4-6) | 172 | (32.7) | 25 | (22.1) | 20 | (23.0) | | 24 | (30.2) | 19 | (19.7) | 32 | (34.4) |
| Some hardship (2-3) | 172 | (32.6) | 36 | (31.3) | 26 | (31.1) | | 34 | (42.7) | 37 | (37.2) | 36 | (38.5) |
| Little hardship (1) | 96 | (18.2) | 18 | (15.9) | 20 | (23.8) | | 10 | (12.7) | 19 | (18.8) | 6 | (6.5) |
| No hardships (0) | 88 | (16.6) | 35 | (30.7) | 19 | (22.1) | | 11 | (14.4) | 24 | (24.2) | 19 | (20.7) |
| ^a^ Lost income included loss of job or reduced hours, or loss of work-related income  ^b^ Unmet expenses included not having enough money to meet daily needs or not enough money to pay monthly bills  ^c^ Debt included using up all/most of savings, having no savings before the pandemic, or having gone into debt or increased debt during the pandemic  ^d^ Unmet healthcare expenses included loss of health insurance, not having enough money to pay for healthcare, and not having enough money to pay for medications  ^e^ Housing insecurity included not having a regular place to live and not having enough money to pay rent, mortgage, or housing costs  ^f^ Food insecurity included being hungry but didn’t eat because not enough money for food  ^g^ Financial hardship index was calculated by counting the number of domains each participant reported experiencing (range 0-6); financial hardship was categorized as dichotomous (≥1 [any] vs. no hardships) and into categories (substantial [4-6], some [2-3], little [1], and no hardships) | | | | | | | | | | | | | |

**Supplemental Table 5.** Prevalence of financial hardship during the pandemic among Latino adults, stratified by heritage, weighted to be nationally representative within racial/ethnic groups, December 2020-Februrary 2021.

|  | **Native Hawaiian** | | **Pacific Islander** | |
| --- | --- | --- | --- | --- |
| **Total, N** | 274 |  | 222 |  |
| **Financial hardship domains, n (%)** |  |  |  |  |
| Lost income^a^ | 130 | (47.5) | 129 | (57.2) |
| Unmet expenses^b^ | 99 | (36.2) | 122 | (55.1) |
| Debt^c^ | 167 | (61.1) | 165 | (72.9) |
| Unmet healthcare expenses^d^ | 64 | (23.4) | 73 | (32.1) |
| Housing insecurity^e^ | 67 | (24.6) | 69 | (30.9) |
| Food insecurity^f^ | 53 | (19.3) | 49 | (22.0) |
| **Financial hardship index^g^, mean (SD)** | 2.12 | (2.0) | 2.69 | (1.8) |
| **Any financial hardship^g^, n (%)** | 205 | (74.7) | 188 | (84.7) |
| **Financial hardship type^g^, n (%)** |  |  |  |  |
| Substantial hardship (4-6) | 71 | (26.0) | 80 | (36.2) |
| Some hardship (2-3) | 72 | (26.3) | 69 | (31.1) |
| Little hardship (1) | 61 | (22.4) | 39 | (17.4) |
| No hardships (0) | 69 | (25.3) | 34 | (15.3) |
| ^a^ Lost income included loss of job or reduced hours, or loss of work-related income  ^b^ Unmet expenses included not having enough money to meet daily needs or not enough money to pay monthly bills  ^c^ Debt included using up all/most of savings, having no savings before the pandemic, or having gone into debt or increased debt during the pandemic  ^d^ Unmet healthcare expenses included loss of health insurance, not having enough money to pay for healthcare, and not having enough money to pay for medications  ^e^ Housing insecurity included not having a regular place to live and not having enough money to pay rent, mortgage, or housing costs  ^f^ Food insecurity included being hungry but didn’t eat because not enough money for food  ^g^ Financial hardship index was calculated by counting the number of domains each participant reported experiencing (range 0-6); financial hardship was categorized as dichotomous (≥1 [any] vs. no hardships) and into categories (substantial [4-6], some [2-3], little [1], and no hardships) | | | | |
